# The impact of episodic future thinking on sports mental resilience and social inclusion among wheelchair tennis players

**DOI:** 10.4102/ajod.v15i0.1819

**Published:** 2026-01-26

**Authors:** Bhuvaneshwar Siva, Om Prakash Palanivel, Kiran Gajendran, Chinnavan Elanchezhian, Stella Rajkumar, Sathish Kumar Sadagobane, Arun Prakash Krishna Moorthy, Charulatha Ganeshkumar, Jeevarathinam Thirumalai

**Affiliations:** 1 Department of Sports Sciences, Saveetha College of Physiotherapy, Saveetha Institute of Medical and Technical Sciences, Chennai, India; 2 Department of Cardiovascular and Pulmonary Sciences, Saveetha College of Physiotherapy, Saveetha Institute of Medical and Technical Sciences, Chennai, India; 3 School of Physiotherapy, Faculty of Allied Health Professions, AIMST University, Kedah, Malaysia; 4 Department of Pediatrics, Saveetha College of Physiotherapy, Saveetha Institute of Medical and Technical Sciences, Chennai, India; 5 Department of Physiotherapy, Faculty of Medicine and Health Sciences, University of Tunku Abdul Rahman, Jalan Sungai Long, Selangor, Malaysia

**Keywords:** episodic future thinking, social inclusion, sports mental resilience, strengthening exercise, visualise, wheelchair tennis player

## Abstract

**Background:**

The intricate process of ensuring equitable access to opportunities, resources and connections through social inclusion promotes social interaction. Resilience is frequently associated with these variances, and the ability to bounce back from setbacks is one of the most crucial components of athletic success. Episodic future thinking (EFT) is the capacity to visualise oneself in the future, establishing a link between one’s present and future selves, promoting resilience and social integration.

**Objectives:**

To evaluate the impact of EFT on sports mental resilience and social inclusion among wheelchair tennis players. To assess the social inclusion and mental resilience of wheelchair tennis players.

**Method:**

This was a randomised controlled trial and employed a convenience sampling technique to recruit 36 wheelchair tennis players. It was divided into two groups: the Experimental group: EFT combined with strengthening exercises and the conventional group: Counselling combined with strengthening exercises. This study was formally registered in the Thai Clinical Trial Registry (TCTR) under the registration number TCTR20250310008.

**Results:**

A total of 36 wheelchair tennis players with findings on Social Inclusion and Sports Mental Resilience were included, with 18 in the experimental group and 18 in the conventional group. Both groups were comparable in post-mean values. The experimental group showed statistically significant results compared to the conventional group (*p* < 0.05).

**Conclusion:**

This study concludes that while both EFT and conventional training improve sports’ mental resilience and social inclusion.

**Contribution:**

The combination of EFT with strengthening exercises is slightly more effective than conventional training.

## Introduction

Wheelchair tennis emerged much later than traditional tennis, which originated in 1874. It was first played in 1976, and within just 8 months, the first wheelchair tennis federation in the United States had over 500 members (Stănescu 2014; Altgassen 2015; Costello, Egger & Angold 2005; Huang & Brittain 2006). Created by Brad Parks, the sport gained international recognition and was added as a Paralympic event at the 1988 Seoul Games. People with physical disabilities often face stigma and discrimination, affecting their social interactions and self-esteem. Disability is commonly understood through two models: the medical model, which treats disability as a personal impairment, and the social model, which emphasises societal barriers (Sivri 2024; Bø et al. 2022; Clutterbuck et al. 2024). Sports have historically been vital in rehabilitation and inclusion. Notably, Dr. Ludwig Guttmann used sports to improve the lives of those with spinal injuries, eventually leading to the Paralympic Games in 1960 (Sánchez-Pay & Sanz-Rivas 2020). Wheelchair tennis uses a standard court and similar rules as able-bodied tennis, promoting equality in competition (Leutar & University of Zagreb 2017).

In sports, episodic future thinking (EFT) boosts motivation by helping athletes imagine future achievements and remain committed to training (Leahey et al. 2020; Asayama et al. 2024; Fari 2023). Episodic future thinking involves visualising future events and has shown success in encouraging positive behaviours like regular exercise and smoking cessation (Daniel et al. 2015; Roetert et al. 2024). Episodic future thinking also enhances social inclusion, particularly in sports like wheelchair tennis, which fosters a sense of belonging and skill development (Richardson et al. 2017). It contributes to sports’ mental resilience by supporting long-term goal setting and stress management (Vella et al. 2021; Hallford et al. 2018).

This study aimed to examine the impact of EFT on sports’ mental resilience and social inclusion among wheelchair tennis players, filling a gap in research and identifying effective strategies to support athletes with disabilities.

## Research methods and design

This study was approved by the Institute Ethics Committee and was registered in the Thai Clinical Trial Registry under registration no. TCTR20250310008. Wheelchair tennis players (aged 18–28) were selected using convenience sampling from a camp organised by the Department of Physical Education, SIMATS Thandalam, Chennai. Inclusion criteria required participants to have a medically verified physical disability, spinal cord injury such as Paraplegia due to Lumbar Spinal Cord Injury (SCI), Paraplegia due to Sacral SCI, Spina Bifida (Myelomeningocele), and basic wheelchair tennis skills. Exclusion criteria: participants having significant cognitive impairments, severe psychiatric conditions, medical conditions such as hypertension that could significantly interfere with participation in the study, and those who do not engage in wheelchair tennis were not included. Participants were randomly assigned to two groups and trained five times per week for 8 weeks. The interventions consisted of strengthening exercises combined with *EFT* for the experimental group, while the control group received counselling combined with strengthening exercises. Primary outcomes were measured using the Social Isolation Scale (SIS) (Blauwet & Willick 2012) and the Sports Mental Toughness Questionnaire (SMTQ) (Roncone et al. 2020). The SIS scores range from 21 to 105, with higher scores indicating greater social inclusion and connectedness: 85–105 reflects strong inclusion, 50–84 indicates moderate inclusion, and 21–49 suggests social isolation. The SMTQ assesses mental toughness on a scale of 14–56, with higher scores reflecting greater confidence, focus and resilience: 43–56 indicates strong mental toughness, 29–42 indicates moderate mental toughness with room for improvement, and 14–28 indicates low mental toughness, suggesting challenges with confidence or emotional control.

The sample size (*n* = 36) was determined using G*Power software, assuming an effect size of 0.99, an alpha level (α) of 0.05, and a power of 0.82 for two groups.

Informed consent was obtained from all participants after explaining the procedures, safety and effectiveness.

### Study procedure

A total of 36 participants who met the eligibility criteria were recruited and completed the study. All participants completed the 8-week intervention and post-assessment, with no dropouts or exclusions. The participant number remained consistent throughout the study. Participants were randomly allocated into two equal groups: Group A (*n* = 18) received EFT with strengthening exercises, and Group B (*n* = 18) received counselling with strengthening exercises. Pre- and post-intervention assessments were conducted using the SIS and the SMTQ. Data were analysed using SPSS 21.

### Protocol

#### Experimental group

Participants in the experimental group received EFT combined with strengthening exercises. Training was conducted five times per week for 8 weeks:

**Week 1:** In the first week, the focus was on introduction and baseline assessment to understand the athletes’ current performance levels, goals and challenges while introducing EFT. Athletes learned about EFT and its benefits for sports performance, along with basic visualisation techniques emphasising sensory details and emotions. As part of the practice, they wrote down three specific and vivid future performance scenarios, such as winning a match or executing a perfect play.

**Week 2:** The main focus of the second week was creating meaningful and in-depth future scenarios related to sports’ goals. By adding sensory information, emotional reactions and contextual components like training sessions or competition days, athletes improved their scenarios. They visualised achievable short-term goals during guided EFT sessions and practised these scenarios for 5–10 min each day, concentrating on one at a time as part of their home routine.

**Week 3:** The third week focused on linking *EFT* to daily training and motivation. Athletes learned the benefits of visualising success and practiced connecting future events to daily routines, such as visualising perfect technique before training. They also participated in role-playing activities to apply EFT in overcoming obstacles. As part of home practice, athletes continued daily EFT exercises and kept a journal detailing how EFT influenced their motivation to train.

**Week 4:** The fourth week emphasised applying *EFT* to improve emotional regulation and stress management during performance. Athletes practised visualising success under pressure and learned relaxation-based EFT techniques to reduce pre-competition anxiety. They used EFT during stressful scenario simulations to prepare for successful outcomes and reflected on their emotional responses during at-home practice.

**Week 5:** The fifth week emphasised developing mental resilience and improving focus through *EFT*. Athletes created resilience scripts for overcoming potential setbacks and explored how EFT helps maintain focus on long-term goals despite challenges. During competitions, they visualised recovering from mistakes and composed resilience-focused EFT scenarios for home practice.

**Week 6:** During the sixth week, athletes were encouraged to incorporate *EFT* into their daily routines as a sustainable habit. They created customised EFT routines for pre-training and pre-competition, evaluated their progress, and refined their scenarios. Athletes also integrated EFT with other mental preparation techniques, such as affirmations and mindfulness. During home practice, they followed their personalised routines and tracked the impact on performance.

**Week 7:** The goal of the seventh week was to enhance *EFT* by adding dynamic components. Athletes incorporated movement and mental rehearsal into their EFT practice, visualising success while mentally rehearsing specific skills. They developed a comprehensive preparation regimen combining physical warm-ups with EFT and tested advanced methods for home practice, recording their effectiveness.

**Week 8:** In the eighth week, the focus was on evaluating progress and developing a long-term *EFT* strategy. Athletes assessed their improvements based on initial goals and received feedback for further development. They created a personalised EFT schedule aligned with competition cycles and implemented the plan as part of their ongoing home practice, monitoring progress quarterly.

### Strengthening exercise (for both experimental and conventional groups)

For wheelchair players, it is crucial to include exercises that strengthen balance and stabilise the posterior upper-body muscle groups. Because pushing motions are more common than pulling motions in both daily and athletic wheelchair use, there is often greater emphasis on anterior upper-body strength. Consequently, these muscles may become overdeveloped and more prone to overuse injuries.

Depending on the degree of impairment, wheelchair tennis players followed tailored strength training regimens. Training was conducted five sessions per week for 8 weeks.

### For athletes with minimal impairments

Recommended exercises included dumbbell biceps curls (2–3 sets of 8–12 reps), triceps pushdowns (2–3 sets of 8–12 reps), pull-ups (2–3 sets of 10–15 reps), inverted rows (2–3 sets of 10–15 reps) and chest cable flys (2–3 sets of 8–12 reps).

### For athletes with more severe impairments (based on International Tennis Federation classification)

Exercises included cable machine or resistance band chest presses (2–3 sets of 8–12 reps), cable or band rows (2–3 sets of 8–12 reps), shoulder lateral raises (2–3 sets of 8–12 reps), band pull-aparts (2–3 sets of 8–12 reps), band biceps curls (2–3 sets of 8–12 reps) and triceps band overhead extensions (2–3 sets of 8–12 reps). Assistive devices were appropriately positioned as needed.

In addition, wheelchair players performed on-court conditioning and movement drills such as the spider drill (1–3 sets of 1–3 reps), backward sprints from baseline to service line and back (2–5 sets of 3–5 reps) and lateral sprints from doubles sideline to sideline (2–5 sets of 4–8 reps).

### Conventional group

Participants in the conventional group received counselling combined with strengthening exercises. General counselling for wheelchair tennis players (10–20 min) was conducted five times per week for 8 weeks.

Counselling sessions focused on managing fatigue, recovery and injury prevention, as well as developing support systems (e.g., coaches, family or friends). Athletes discussed balancing tennis with personal life, setting short- and long-term goals, handling pressure during crucial matches, staying motivated after setbacks and identifying areas for improvement in wheelchair tennis. Sessions also addressed mental and physical preparation strategies for competition.

### Statistical analysis

The data were analysed using means and standard deviations. Baseline comparisons revealed a significant difference between the experimental and conventional groups for some pre-test measures. Therefore, we performed Analysis of Covariance (ANCOVA) to compare the post-test results, adjusting for baseline differences. Paired *t*-tests assessed pre- and post-test differences within groups, while unpaired *t*-tests compared differences between groups. A *p*-value < 0.05 was considered statistically significant.

### Ethical considerations

Ethical clearance to conduct this study was obtained from the Institutional Scientific Review Board on Human Subjects, Saveetha College of Physiotherapy, Saveetha Institute of Medical and Technical Sciences (No. 212/07/2024/ISRB/UGSR/SCPT).

## Results

The demographic characteristics of the participants are presented (Table 1).

**TABLE 1:** Participants’ demographic data (*N* = 36).

Variable	Category	Experimental group	Conventional group	*n*
Gender	Male	11	10	36
	Female	07	08	-
Age (years)	18–20	04	05	09
	21–24	06	06	12
	25–28	08	07	15
Body Mass Index (BMI, kg/m^2^)	16.5–18.5 (Underweight)	03	03	06
	19.5–24.9 (Normal weight)	10	11	21
	25–29.9 (Overweight)	05	04	09

Social Isolation scores significantly improved in the EFT group (pre = 63.94 ± 6.83, post = 88.06 ± 5.92, *t* = –17.456, *p* < 0.001) and in the counselling group (pre = 50.11 ± 10.12, post = 67.22 ± 6.19, *t* = –11.315, *p* < 0.001) (Table 2).

**TABLE 2:** Pre-test and post-test value of Social Inclusion Scale (*N* = 36).

Outcome measure	Test	Mean	s.d.	Adjusted post-test		F (1, 34)	ANCOVA *p*-value	*t*	*p*-value
				Mean	s.e.				
Experimental	Pre-test	63.94	6.83	-	-	-	-	-	-
	Post-test	88.06	5.92	82.26	1.17	-	< 0.001	−17.456	< 0.001
Conventional	Pre-test	50.11	10.12	-	-	4.304	-	-	-
	Post-test	67.22	6.19	55.23	1.17	-	< 0.001	−11.315	< 0.001

s.d., standard deviation; s.e., standard error; ANCOVA, Analysis of Covariance; F, Fisher’s F-statistic.

Sports Mental Toughness scores also improved significantly in both groups: EFT group (pre = 36.61 ± 4.53, post = 47.94 ± 4.68, *t* = –13.427, *p* < 0.001) and counselling group (pre = 29.22 ± 5.95, post = 32.22 ± 5.35, *t* = –8.746, *p* < 0.001) (Table 3).

**TABLE 3:** Pre-test and post-test value of Sports Mental Toughness Questionnaire (*N* = 36).

Outcome measure	Test	Mean	s.d.	Adjusted post-test		F (1, 33)	ANCOVA *p*-value	*t*	*p*-value
				Mean	s.e.				
Experimental	Pre-test	36.61	4.52	-	-	-	-	-	-
	Post-test	47.94	4.68	44.91	0.68	-	0.001	−13.427	< 0.001
Conventional	Pre-test	29.22	5.95	-	-	82.07	-	-	-
	Post-test	32.22	5.35	35.24	0.68	-	< 0.001	−8.746	< 0.001

s.d., standard deviation; s.e., standard error; ANCOVA, Analysis of Covariance; F, Fisher’s F-statistic.

The post-test results revealed significant improvements in both outcome measures for the group that received EFT combined with Strengthening Exercise compared to counselling combined with Strengthening Exercise. In the SIS, the EFT group showed a mean ± s.d. of 47.94 ± 4.68, while the counselling group recorded 32.22 ± 5.35 (*t* = 9.378, *p* < 0.001). Similarly, in the SMTQ, the EFT group achieved 88.06 ± 5.92 versus 67.22 ± 6.19 in the counselling group (*t* = 10.324, *p* < 0.001). These findings indicate that EFT combined with Strengthening Exercise is significantly more effective than counselling combined with Strengthening Exercise in enhancing social inclusion and mental toughness among wheelchair tennis players (Table 4).

**TABLE 4:** Post values of experimental and conventional group (*N* = 36).

Outcome measure	Group	Mean	s.d.	*t*	*p*-value
SIS	Experimental group	88.06	5.92	10.324	< 0.001
	Conventional group	67.22	6.19	-	-
SMTQ	Experimental group	47.94	4.68	9.378	< 0.001
	Conventional group	32.22	5.35	-	-

SIS, social isolation scale; SMTQ, sports mental toughness questionnaire; s.d., standard deviation.

These findings confirm that EFT combined with strengthening exercises was more effective than counselling with strengthening.

## Discussion

This study explored the effects of EFT with strengthening exercises on social inclusion and mental resilience in 36 wheelchair tennis players (aged 18–28). Over 8 weeks, Group A received EFT with strengthening, while Group B received counselling with strengthening. Outcomes were assessed pre- and post-intervention using the SIS and SMTQ.

Anderson et al. (2024) found that episodic simulation can strengthen implicit positive expectations about the future. Their study supports the use of repeated EFT to promote optimism and suggests its potential in developing interventions for mental health, particularly depression (Anderson et al. 2024).

This study demonstrates statistically significant improvements in sports mental resilience and social inclusion among wheelchair tennis players with Group A who received EFT combined with strengthening exercises showing greater progress than Group B. Consequently, Group A outperformed Group B in both sports’ mental resilience and social inclusion, highlighting the effectiveness of integrating EFT with strengthening exercise.

### Limitations of the study

The study’s limitations include a small sample size (*n* = 36), an 8-week duration that prevented long-term assessment, and no follow-up to evaluate sustained EFT effects. Pain duration was not measured, which may have influenced resilience, and objective performance outcomes were not assessed, limiting insight into EFT’s impact on actual competition.

## Conclusion

The study concludes that while both interventions improved sports mental resilience and social inclusion, EFT combined with strengthening exercises was slightly more effective. Further research with long-term follow-up is needed to confirm these findings and evaluate sustained benefits.
